# Living with Urinary Incontinence: Potential Risks of Women’s Health? A Qualitative Study on the Perspectives of Female Patients Seeking Care for the First Time in a Specialized Center

**DOI:** 10.3390/ijerph16193781

**Published:** 2019-10-08

**Authors:** María Zahara Pintos-Díaz, Cristina Alonso-Blanco, Paula Parás-Bravo, César Fernández-de-las-Peñas, María Paz-Zulueta, Víctor Fradejas-Sastre, Domingo Palacios-Ceña

**Affiliations:** 1 Department of Rehabilitation, Hospital 12 Octubre, Madrid Health Service, Avda, Cordoba, 28041 Madrid, Spain; zahara.pintos@salud.madrid.org; 2 Department of Physiotherapy, Occupational Therapy, Rehabilitation, and Physical Medicine, University Rey Juan Carlos, Avda de Atenas, Alcorcón, 28922 Madrid, Spain; cristina.alonso@urjc.es (C.A.-B.); cesar.fernandez@urjc.es (C.F.-d.-l.-P.); domingo.palacios@urjc.es (D.P.-C.); 3 Department of Nursing, Faculty of Nursing, University of Cantabria, Avda Valdecilla, Santander, 39008 Cantabria, Spain; maria.paz@unican.es (M.P.-Z.); victor.fradejas@unican.es (V.F.-S.); 4 Researh Nursing Group IDIVAL, Calle Cardenal Herrera Oria s/n. C.P., 3901 Cantabria, Spain; 5 Health Law and Bioethics Group, Health Research Institute IDIVAL, c/Cardenal Herrera Oria s/n, 39011 Santander, Cantabria, Spain; 6 Research Group of Humanities and Qualitative Research in Health Science of Universidad Rey Juan Carlos (Hum&QRinHS), University Rey Juan Carlos, Avda de Atenas, Alcorcón, 28922 Madrid, Spain

**Keywords:** urinary incontinence, qualitative study, health education, women

## Abstract

Background: Urinary incontinence (UI) represents a complex problem which commonly affects women and influences their physical, mental, and social wellbeing. The objective of this study was to explore the experiences of a group of women with urinary incontinence. Methods: A qualitative exploratory study. Purposeful sampling was used. Recruited patients were females aged >18 years old with positive symptoms, signs of urinary incontinence, and attending a specialized urinary incontinence center for the first time. We collected data using interviews and participants’ personal letters. A thematic analysis was performed. Results: 18 women participated with a mean age of 47.32 years. Four themes emerged: (a) Experiencing uncontrolled urinary leakage, (b) Information based on beliefs and myths regarding UI, (c) Adapting to change and developing strategies, (d) The role of education. Women’s experiences were accompanied by feelings of stress and shame. A lack of information regarding UI was found, together with numerous misconceptions. Urinary incontinence triggers many changes in women. Some women may develop feelings of rejection towards their own body. Family involvement during these times is essential for education and promoting healthy sexual practices. Conclusions: This study highlights the importance of developing educational programs that focus on women’s information and education regarding triggering factors and coping strategies.

## 1. Introduction

Urinary incontinence (UI) or involuntary leakage of urine [1] is a distressing health problem [2]. The three most common types of UI are stress urinary incontinence (SUI), urge urinary incontinence (UUI), or a combination of both, i.e., mixed urinary incontinence (MUI) [2]. First line management includes lifestyle and behavior modification, as well as pelvic floor strengthening and bladder training. This is linked to other treatments, depending on the type of UI, such as pharmacological therapy and surgery [3]. Nearly 50% of adult women (<60 years) may experience UI in their lifetime. Furthermore, UI increases with age, affecting 10% to 20% of elderly women [4]. Moreover, there is an overall prevalence of 17% among women aged 20 years or older and 38% among women aged 60 years or older [4]. Despite this high prevalence, UI remains underdiagnosed and undertreated [5]. It is estimated that 25–37.3% of affected women seek care and, of these, less than half receive treatment [4,6].

Urinary incontinence is associated with increased rates and severity of female sexual dysfunction, such as: impaired body image, fear of coital UI, avoidance of sex, decreased desire, lubrication, and satisfaction, as well as increased pain [3]. Moreover, untreated incontinence is associated with falls and fractures, sleep disturbances, depression, and urinary tract infections [4,7,8]. This problem constitutes an important psychosocial and economic burden, with significant consequences on quality of life [9]. Individual perspectives and the narratives concerning UI are as relevant as much as one’s life experiences, and emotional needs must be considered when treating the individual [10]. Previous studies have reported how UI affects women’s daily activities, their social roles, and their feelings of intimacy and sexual satisfaction [10], as well as their expectations of pelvic floor rehabilitation [11,12]. Furthermore, some authors have studied the cultural context in which women experience these changes [13,14]. Moreover, UI is related to the presence of perceived stigma. This is defined as a label that associates a person to a set of unwanted characteristics that changes behavior and shapes their emotions and beliefs [15]. 

Stigma enhances the formation of negative attitudes towards seeking professional help for urinary incontinence through the effect of internalized shame [15,16,17]. Other factors that influence the delay in consulting medical professionals or seeking help are: the presence of preconceived ideas and myths about UI and its inevitability as part of a woman’s life cycle [18,19]; difficulty in accessing specialized care [18]; fear that professionals do not give importance to UI or play down the loss of urine [18,19]; not being specifically asked about incontinence [19]; not knowing the language of the host country and the fear of being criticized for having different beliefs and culture/ethnicity (in the case of immigrant women) [16,20]; and demonstrating different abilities and coping skills [16].

Likewise, Norton et al. [16] pointed out that these non-biological factors are necessary in order to understand the UI phenomenon in women. Additionally, a comprehensive, interdisciplinary approach to research is needed to appropriately support effective UI treatment, help women to seek care, and identify barriers or facilitators to seek help. Previous studies have reported barriers which either delayed or made it more difficult to seek professional help for women with UI [16,19,21]. However, few qualitative studies [10,22] reported the participants’ responses to the symptoms and to UI. Welch et al. [22] described patient-reported outcomes with lower urinary tract symptoms for UI and most patients experienced either no symptom relief or partial relief. Likewise, Mendes et al. [10] showed how cultural and religious backgrounds contribute to delays in seeking UI treatment in adult women. To the best of our knowledge there are no qualitative studies that explored the experiences of women with UI to understand what triggers them to seek care in a specialized center of UI for the first time. Therefore, the research questions of this qualitative study were: what is the experience of women with UI who seek care for first time? What reasons do women have for seeking specialized care for the first time? What factors act as facilitators or hindrances for seeking help? Thus, the purposes of this study were: a) to identify the experience of a group of Spanish women diagnosed with UI who sought care for the first time in a specialized center, and b) to describe the reasons that trigger the search for help for the first time. 

## 2. Materials and Methods

The present study forms part of a larger research project which objective was to study new pelvic floor rehabilitation therapies, and the impact of urinary incontinence in the women´s life. In the first quantitative research phase, previously published, we used a non-randomized clinical trial involving 60 women >18 years of age, both with and without urinary incontinence [23]. The objective was to determine the effect of pelvic floor muscle training using a tampon as visual biofeedback [23]. The second phase of the project, a qualitative exploratory design, aims to study the experience and perspective of women with UI. Mixed methods (quantitative and qualitative designs) to analyze complex realities have often been used in health sciences [24,25].

### 2.1. Design

A qualitative exploratory and descriptive study [26,27] was carried out to identify the experiences of Spanish women with UI seeking help for the first time. In qualitative research, a qualitative descriptive study portrays a comprehensive summary of events using the everyday terms of those events. In this type of design, researchers stay close to their data and to the surface of words and events. Concretely, qualitative exploratory and descriptive study is recommended when straight descriptions of phenomena are desired [26,27]. Combinations of sampling, data collection, and analysis are typically used in qualitative exploratory and descriptive study [26]. In the present qualitative exploratory study, two key assumptions about the study’s descriptive approach were applied: (1) there are features in any experience that are common to anyone who has had that experience; and (2) it is important for the researcher to adopt and maintain an open attitude throughout the research process by “bracketing” or setting aside any preconceived opinions. The use of bracketing allows a critical examination of the phenomena without influencing the researcher’s own beliefs [28]. One bracketing condition was established in the current study: the performance of unstructured interviews without a predetermined questions guide [28]. Bracketing was established during data collection, to avoid researchers asking questions aimed at their own experience and knowledge rather than based on the experiences of participants [28]. Applying bracketing via unstructured interviews, with opening questions and without the use of a questions guide, enabled participants to freely provide information about their experience. 

### 2.2. Research Team

Prior to the study, the position of the researchers was established according to their previous experience [29]. Four researchers (MZPD, CAB, VFS, and MPZ) had clinical and research experience in UI and pelvic floor disorders (PFD). The remaining researchers (DPC, PPB, and CFP) had experience in qualitative study designs, were not involved in clinical activity, and had no prior relation with the participants.

### 2.3. Setting

The setting for this study was a specialized UI center in Madrid (Spain) performing assessments, treatments, follow-up, and specialized care of women with PFD. The center’s professional team was formed by gynecologists, nurses, midwives, and physiotherapists who are experts in PFD and urinary incontinence. The pelvic floor assessment was carried out based on both a physical and an electromyographic (EMG) examination. The center was fully equipped with technical equipment, such as the Verity Medical Neurotrac® (Verity Medical Ltd, Romsey, UK) [30], which was the device used to provide the EMG Biofeedback (BFB), as well as the necessary software [31] computers, and consumables (such as vaginal and anal probes, skin electrodes, disposable gloves, and lubricant), required in order to perform an effective and thorough assessment and treatment of those women with PFD.

### 2.4. Participants and Sampling Strategies

Patients with UI who attended the specialized UI center (Madrid, Spain) for the first time were enrolled in the study between June 2015 and June 2017. Purposeful sampling methods were employed based on the relevance to the research question (not based on clinical representativeness). All patients were recruited during their first visit to the clinic to ensure that none had experienced a significant improvement at the time of the study. The recruitment took place when the women initially visited the clinic. If they met the inclusion criteria and agreed to participate, then they were included in the research project. During this first visit, they were given information on the study and participation in the study was confirmed two weeks later by telephone. The recruitment was performed by MZPD. The women who agreed to participate were consecutively enrolled in the study. The interviews were scheduled during the following two weeks. The sampling process continued until the researcher achieved information redundancy [29], at which point no new information emerged from the data analysis (in our study, this occurred with participant 18). No participants withdrew from the study.

The subjects included in the study were: (a) females aged >18 years old; (b) who attended the center due to an involuntary loss of urine; (c) with UI symptoms and signs according to the American Urological Association and Society of Urodynamics, Female Pelvic Medicine guidelines [32], for example stress, urgency, coital urinary incontinence, nocturnal enuresis, extraurethral incontinence, etc. The exclusion criteria included: (a) a pregnancy and/or post-natal situation (up to three months after delivery); (b) pelvic organ prolapse; (c) persistent urinary tract infections; (d) serious systemic and/or psychiatric disorders; (e) recipients of physical therapy interventions for the pelvic area over the previous year; and (f) inability to communicate in Spanish or to sign the informed written consent form. 

### 2.5. Data Collection

Prior to data collection, two briefing sessions were conducted to determine the data collection procedure (questions to be asked during the course of the interview, interview location, identification of participants, etc.), and to confirm that the process of data collection was known by the entire research team. First-person data collection tools (in-depth interviews and participants’ letters) were used. Subsequently, researcher field notes were obtained [29]. Open-ended interviews were used as the main tool for data collection. These were minimally structured and without time limitations. After establishing rapport, the initial questions asked participants to describe their experience in as much detail as possible. The interviews started with an open question: ‘What is your experience with UI?’ What are the reasons that made you seek help for the first time? Thereafter, the researchers listened carefully, noted the key words and topics identified in the females’ responses, and used their answers to clarify the content [29]. The following questions focused on points of elaboration and clarification. Reflective statements are used to encourage participants to describe the event or experience things in more detail. Verification can occur by restating earlier parts of the conversation. The interviews took place in a private room at the UI center and were conducted by a female researcher (MZPD). All women were interviewed alone. The interviews were conducted in Spanish.

The women participating in the study were asked to write personal letters, which were used as part of the analyzed data, bringing the task of data collection into the respondents’ everyday world [33]. These letters were meant to obtain information in a non-obstructive manner by capturing ordinary events and observations that might be neglected by single-recording methods because often participants perceive these as being insignificant, or fail to remember them [33,34]. Participants were given a two-week period to respond to the same questions used in the interviews [34]. This procedure sought to avoid affecting how the participant conceptualized their responses, and perceived events. The participants were requested to describe the most relevant aspects of their experience according to their perspective, with no limits to the length of the accounts nor the contents of the same [33,34]. Participants were recommended to write at least once a day, whenever they chose to do so. The written accounts were subsequently handed in either in paper or electronically, according to their personal preference [34].

The field notes were collected during the interviews with the aim of noting participants’ gestures and non-verbal language (for example: nervousness or discomfort). The researcher also collected descriptions of the place where the interviews were held, comments on methodological aspects and descriptions of any event (for example: interruptions during the interview) taking place during data collection.

All the interviews were digitally audio recorded and transcribed verbatim (MZPD, CAB, CFP, MPZ, VFS, DPC, and PPB). In total, 1450 min of interviews were recorded and the average time of each interview was 80.55 minutes. The full literal transcription of each of the interviews, the women’s letters and the researchers’ field notes were all collated in order to perform a qualitative analysis. 

### 2.6. Data Analysis

A thematic analysis was performed, which began by analyzing the most descriptive content in order to reach meaningful units. This then went into further depth to produce thematic code groups (i.e., grouping meaningful units referring to the same issue or with the same content until the main topics emerged). To identify the relevant content, researchers read and reread the data at three different levels: literally, interpretively, and reflexively [29]. In this manner, an increasing level of abstraction and complexity was established for the analysis from meaningful units to thematic code groups and, finally, themes [35]. Immediately after each interview, data analysis procedures began. Each interview was analyzed by two different members of the research team (MZPD, DPC). The analysis of the women´s letters were integrated into a matrix, together with the analysis of the interviews. 

Subsequently, meetings were held to identify and compare the results obtained. The final outcome was the identification of themes that represented the women’s experiences with UI who sought care for first time. No qualitative software was used to analyze the data. Data was classified, categorized, and organized using Microsoft Excel.

### 2.7. Rigor

The guidelines for conducting qualitative studies established by the Consolidated Criteria for Reporting Qualitative Research (COREQ) [36] and the Standards for Reporting Qualitative Research (SRQR) [37] were followed. Furthermore, we used criteria by Lincoln and Guba [38,39] for establishing trustworthiness of the data by reviewing issues concerning the credibility, transferability, dependability, and confirmability of the data (Table 1).

### 2.8. Ethics

The study was approved, and all procedures were followed in accordance with the ethical standards of the Ethics Committee of the University Rey Juan Carlos, Spain (code: 20/2015). Informed consent and permission to record the interviews was obtained from all participants included in the study.

## 3. Results 

Eighteen females with SUI (61.11%), UUI (22.22%), and MUI (16.66%) were enrolled in this study, and the mean age of participants was 47.32 years. Table 2 displays demographic data of participants.

Upon analysis of the data collected, four specific themes emerged which described the experiences of the women with UI and can be used to help women seek specialized help for the first time: (a) Experiencing uncontrolled urinary leakage, (b) Information based on beliefs and myths regarding UI, (c) Adapting to change and developing strategies, and (d) The role of education. In order to facilitate the traceability and identification of the results obtained, these are accompanied below by excerpts of transcripts.

### 3.1. Theme 1: Experiencing Uncontrolled Urinary Leakage

All the participants acknowledged that the negative experiences derived from the loss of urine had forced them to seek help at a specialized urinary incontinence center. The women described the leakage of urine as being uncomfortable, unpleasant, and stressful. Most participants described how UI made them feel unclean. Furthermore, UI triggered feelings of shame, uneasiness, insecurity, suffering, lack of control, blame and low self-esteem. Some women described feeling like they had a “defect” or “fault” making them feel less of a person, to the point of feeling guilty for having UI. Many women were also afraid that the leakage of urine would cause their clothes to smell, so they were continuously checking whether they smelled of urine or if there was a disagreeable odor. Table 3 displays participants’ narratives from this theme.

### 3.2. Theme 2: Information based on Beliefs and Myths Regarding UI

All the women who participated in the study complained about the overall lack of information and about how the little information was available to them was unclear. Many had not heard of UI before suffering the initial symptoms and were unaware of where to go to receive treatment. Furthermore, for many participants, their knowledge of UI was based on a series of beliefs or myths. For example, there was a belief that urinary leakage was a ‘normal’ occurrence which, sooner or later, would affect all women. Another belief was that it worsened with age and that women should resign themselves to having to live with the condition. Another common myth was that UI happened to all women after childbirth, therefore relating urinary leakage to the process of reproduction (pregnancy, giving birth and maternity). Most participants confirmed how this idea was reinforced by their close friends and family and even by some health professionals. Other participants believed that UI was something they had inherited, as there were previous cases in the family. Finally, all the participants expressed how the lack of credible information caused them to have doubts, until they eventually decided to seek help in order to receive trustworthy information. Table 4 displays participants’ narratives based on this theme.

### 3.3. Theme 3: Adapting to Change and Developing Strategies

Before seeking help, the participants described feeling forced to modify their habits and routines, and how this negatively affected their daily life, for example, by influencing the way they dressed or their social life. Women described feeling forced to develop strategies to manage the urinary leaks and avoid so-called ‘accidents’. One of the participants spoke of having to carefully choose the places she went to and the necessity of always locating the bathroom when she visited new places. Another strategy was to avoid traveling, or limiting any social life in fear of having a urinary leak. Even the use of public transport was seen as a challenge and cause of insecurity. All participants agreed that the most common strategy was to use incontinence pads. Women viewed these as something that gave them a sense of security, ensuring that their clothes would stay dry yet discreet enough to make others unaware of any leakage. Furthermore, some participants said that they wore pads, despite not having heavy leakage, but as a means of protection. Another strategy was the use of fragrances and perfumes, sprayed onto the body and clothes, to avoid any urine smell that may otherwise be filtered through the sanitary pads and/or diapers. Finally, our participants acknowledged that, over time, they felt unable to continue with these strategies and therefore decided to seek help to control and/or treat their UI. Table 5 displays participants’ narratives from this theme.

### 3.4. Theme 4: The Role of Education

The participants emphasized how the education that they had received at home or the fact of living in a rigid and restrictive social environment had conditioned their experience of UI and how they sought UI care. Family education was seen as being key for the acceptance and recognition of UI and for acquiring healthy hygiene and urinary habits, as well for the development of a healthy sexuality. Sexual education within the family is essential for helping women develop their sexuality. For some women, this experience was natural and positive because there was freedom to discuss and pose questions about the urogenital area and sex within the family. In contrast, others acknowledged feelings of rejection towards the urogenital area, sex, and their sexuality due to the fact that, during their adolescence, their education was driven by fear, thus conditioning their sexual relationships. As such, the social environment of women can clearly shape their experience. Thus, some women spoke about how living in a restrictive, chauvinistic, and paternalistic environment from an early age had influenced them in how they perceived and experienced matters related to their pelvic and genital region, highlighting connotations of sin, guilt, and danger. These areas were perceived as being taboo, associated with many misconceptions, and a considerable emotional burden. After attending a specialized center, the women experienced a change in how they perceived this region. Some women felt that their urogenital region defined them as a woman, playing an important role in their personal story. Other women felt they had rediscovered their intimate body parts through the exercises and techniques that they had learned at the specialized center. Table 6 displays participants’ narratives from this theme.

## 4. Discussion

Our results illustrate how UI is experienced with feelings of unpleasantness, shame, insecurity, and guilt. There is a lack of information on UI, and the information available is unclear. Furthermore, there are many false beliefs surrounding this issue. Urinary incontinence triggers changes which force women to develop certain strategies in their daily life. Some women have a negative perception of the pelvic region. Finally, education on behalf of the family and their social environment is necessary in order to acquire healthy hygienic and urinary habits. 

For women, UI was unpleasant, stressful, and traumatic; it led to feelings of shame, insecurity, suffering and guilt, to the extent that some participants described feeling ‘dirty’. Previous research [10,40] has reported how urinary leaks, and issues related to the smell of urine, can lead to feelings of shame, loss of self-esteem, isolation and delay in the search for help, experiencing UI as a ‘stain in life’ [13]. Furthermore, the feeling of shame is greater during sexual relationships and/or in the event of urinary leaks in public [41]. Consequently, self-esteem can become hampered, and women may reject their own body by avoiding personal hygiene habits and attempting to hide their situation [10]. Furthermore, Hägglund and Ahlström [42] reported that, in the case of women who suffer UI, being in a vulnerable situation means that they have no control over UI, living with a body over which they have lost control, and experiencing feelings of powerlessness.

The lack of information on UI and pelvic floor dysfunctions has led to many myths and misconceptions which have strengthened the taboo of UI [18]. This is not a subject that women feel they should speak about and, therefore, those who suffer from UI may display apathy or even deny the problem altogether [43]. Kendall and Bunn [44] have highlighted how, at times, poor communication, the lack of clear education, and the power of experiences were barriers to seeking help because they promote normalization of suffering in silence and therefore disempower women. In addition, health professionals were encouraged to improve communication and education, which could reduce barriers and enable female patients to seek help. Moreover, the authors postulate that the access to informal sources of information (friends, families), or uncontrolled information sources (internet), and the absence of a credible and proven filter, in the women with UI may cause an excess of contradictory information in the women with UI, thus triggering their search for professional help. 

Previous research [10,11,13] has reported that UI leads to a disruption of daily life, has negative effects on women’s intimacy and leads to changes in the ways that women experience their sexuality. As a result, women may self-impose a series of restrictions, meaning the suppression of pleasures and needs and the avoidance of social interactions (dietary restrictions, dress restrictions, decreased physical/leisure activities, restricted travelling, etc.). Therefore the UI implies a negative impact on the quality and frequency of sexual activity [45]. In addition, feelings of despair are described that are related to a feeling of gloom about the future due to living with UI, as well as a sense of ambiguity, and hopelessness [12,13]. Delarmelindo et al. [11] described how women with UI present a mixed state of uncertainty and suffering, together with hope in the effectiveness of the treatment and the rehabilitation of continence. The role of the professional specialized in UI is to accompany women during their rehabilitation process, by informing, guiding and applying interventions based on clear and realistic goals [1]. Additionally, the race/ethnicity, culture and beliefs may be factors that determine the strategies adopted by women [10]. Thus, Siddiqui et al. [14], described how white, black, and Latino women shared experiences of embarrassment and isolation because of UI, plus communication barriers with doctors, as well as reporting the longest delays in seeking care. However, we believe that one of the reasons for seeking help is because of the failure of previous strategies, and also because women hope to be able to learn and develop strategies that are helpful and which may be effective for the control of UI in the long term. Unfortunately, although these may work for a certain time, they fail to offer a long-term solution for the UI and may even cause restrictions to their lifestyle. The strategies developed by our participants are an instrument to try to solve a long-term problem which causes significant limitations in their lives. In the same way, Waetjen et al. [19] and Volkmer et al. [18] reported how women with UI who were more interested in seeking care were older and had experienced urinary leakage for an extended period of time.

Our findings highlight how women who have had negative experiences with their pelvic region describe rediscovering their body when seeking for help and initiating physiotherapy treatment. Kao et al. [12] noted that sex is a taboo topic for many Taiwanese women, and reported how pelvic floor muscle exercises had a positive effect on urinary incontinence and sexuality. Many women ignore the specific features of the pelvic region and the manual therapy intervention facilitated the acceptance of their pelvic region. In our study, the participants were evaluated both manually and electromyographically. In addition, they had to carry out daily training exercises using their pelvic floor muscles with a disposable tampon as a home biofeedback device during the quantitative intervention. This helped them to improve their pelvic floor proprioception and rediscover their bodies. On the other hand, we were unable to find any research analyzing the perceptions of women with UI regarding the function of the pelvic floor. The authors believe that for our participants, the urogenital region and the ability to control urine, equals health (absence of urine leakage), normality (they are like the rest of women), and a reconnection with other aspects of their lives through this region (sexuality). This would be an interesting area for future research as how this meaning is constructed may influence the experience and the way women perceive the illness and symptoms. Health professionals normally refer to the “pelvic floor” in the context of disorders. This orientation presents a ‘pathological’ connotation of a key anatomic region in the vital reproductive-sexual process of the woman. Perce, Perry, Gallagher, and Chiarelli [46] defend the use of the “pelvic floor health” concept to eliminate negative connotations and promote both primary prevention and education.

Finally, our findings highlight how the family and the surrounding context condition a woman’s experience of UI and sexuality. Previous authors [3,12] agree that the effects on women’s intimacy and sexual satisfaction are among most significant consequences of UI. This provokes changes in the ways women experience their urogenital area, sexuality, and sexual function. Furthermore, the society, culture or ethnicity at large often see UI as a disorder where the woman is held responsible, triggering situations of social vulnerability, discrimination and isolation [14]. From our point of view, the family can play an important role by providing support to women with UI. The specialized UI professional therefore must act as the health provider or educator of women and family members, in order to promote health and prevent UI in women throughout their life cycle. Smith et al. [47] reported that specialized UI professionals (women’s health providers and educators), seek post-professional training with an emphasis on pelvic floor assessment and treatment, obstetrics and gynecology, urogenital concerns, complications of cancer, wellness and health promotion, and research.

## 5. Limitations

This study has some limitations concerning generalizability that limit the extrapolation of our results to the whole population with UI. It is important to consider a possible Spanish cultural influence to the themes generated by this research. In this sense, Siddiqui et al. [14] reported there are different perceptions about care seeking among white, black, and Latina women. While white and black women described discussions with close friends that led to normalization of symptoms and avoided care seeking, Latina women maintained more secrecy about UI and reported the longest delays in seeking care. Likewise, Jackson, Hernandez, Mallett, and Montoya [48] described how Spanish-speaking Latinas perceived UI as an abnormal condition, leading them to ignore symptoms of UI, and experiencing fear of doctors as well as feelings of embarrassment. Both situations (normalization and feelings of embarrassment.) mean that women delay seeking help from healthcare professionals.

Additionally, this study was performed on women attending a specialized UI center for the first time; therefore, our findings do not extend to patients with UI who have not visited a UI clinic or specialist. Lastly, the findings of this qualitative descriptive study have been organized to display the experience of the participants, rather than the phenomenon of UI. The findings could have therefore been presented in a different manner. The choice of how to present and order research findings facilitates the understanding of the participants’ experiences and is typical of qualitative exploratory studies [26]. 

## 6. Conclusions

This study provides insight on how UI is experienced by a group of Spanish women who are seeking care at a specialized UI center for first time. Our findings shed light on how UI influences the lives of female patients and triggers the visit to a specialized center for the first time. Our participants’ experiences of living with UI revealed the following: (a) a lack of control over their bodies and uncontrolled and unscheduled urine losses, (b) most of the information on urine loss available is based on myths and beliefs, which raises concerns over the credibility and veracity of the information that the woman with UI receives, (c) the strategies that the women use for the management of urine loss over time are insufficient, so that UI still has a negative impact on their lives, and (d) the women experience a lack of education concerning the urogenital region and on related aspects such as hygiene and sexuality.

Women should receive accurate information regarding UI and the urogenital region from healthcare professionals in order to change false beliefs. Furthermore, women should receive support in order to be able to anatomically recognize their pelvic and urogenital regions. Healthcare professionals can help women to recognize and accept their body. This implies the implementation of educational and training programs on coping and managing urinary incontinence by interdisciplinary teams led by healthcare professionals in different contexts, such as the community, health centers, and UI associations. Similarly, work teams should be formed between healthcare professionals working in the community and education professionals to develop programs in schools and high schools on the promotion of regional urogenital health in girls and adolescents. Furthermore, providers of women’s healthcare services have an important role in the education of women during routine health visits. As such, routine health visits represent an ideal opportunity to provide a unified education, shared by all women’s health care providers (nurses, midwives, doctors, etc.) by providing truthful information on the woman’s body, dispelling myths and false beliefs on UI, clarifying any questions on pelvic and urogenital regions, and promoting healthy hygiene habits. 

Our study also provides grounds to guide healthcare professionals to further research the quality of life of women with UI. Concretely, it would be interesting to analyze the experience of women who have undergone specific treatments for UI in their own contexts, and to identify the factors that helped facilitate the same or that represented barriers for seeking care.

## Figures and Tables

**Table 1:** Trustworthiness criteria.

Criteria	Techniques Performed and Application Procedures
**Credibility**	Triangulation of data collection methods: open-ended interviews were conducted, personal letters were obtained, and researcher field notes were kept. Participant validation: this consisted of asking the participants to confirm the data obtained during the stages of data collection and analysis.
**Transferability**	In-depth descriptions of the study were performed, providing details of the characteristics of researchers, participants, contexts, sampling strategies, and the data collection and analysis procedures.
**Dependability **	Audit by an external researcher: an external researcher assessed the study research protocol, focusing on aspects concerning the methods applied and study design.
**Confirmability**	Data collection triangulation, and participant validation. Researcher reflexivity was encouraged via the performance of reflexive reports and by describing the rationale behind the study.

**Table 2:** Participant demographic data.

Participant	Age	Job	Civil Status	Children	UI Type	Time Patient has had Symptoms (months)	Management Prior to Visiting Specialized Clinic	Themes Endorsed
1	45	Housewife	Married	2	MUI	23	Surgery	T1,T2,T3,T4
2	45	Businesswoman	Married	2	SUI	18	Physiotherapy	T1,T2,T3
3	45	Civil servant	Married	2	MUI	40	None	T1,T2,T3,T4
4	23	Student	Single	1	SUI	1	Yoga	T1,T2,T3
5	45	Housewife	Married	2	UUI	2	Pilates	T2,T3,T4
6	44	Housewife	Married	2	MUI	26	None	T1,T2,T3,T4
7	53	Housewife	Married	1	SUI	30	General Physician	T2,T3,T4
8	51	Housewife	Married	1	SUI	18	None	T2,T3,T4
9	58	Housewife	Married	0	UUI	1	None	T1,T2,T3,T4
10	47	Civil servant	Single	0	SUI	1	General Physician	T1,T2,T3,T4
11	47	Civil servant	Single	1	SUI	2	None	T1,T2,T3
12	48	Manager	Single	0	SUI	46	Physiotherapy	T1,T2,T3
13	47	Housewife	Married	1	SUI	20	None	T1,T2,T3,T4
14	56	Housewife	Married	1	SUI	35	Physiotherapy	T2,T3,T4
15	49	Businesswoman	Single	1	UUI	1	General Physician	T1,T2,T3
16	44	Businesswoman	Single	1	UUI	1	General Physician	T1,T2,T3
17	49	Civil servant	Single	1	SUI	42	None	T1,T2,T3
18	56	Manager	Single	2	SUI	17	Pilates	T2,T3,T4

Note: MUI= mixed urinary incontinence; T1=theme 1; T2=theme 2; T3=theme 3; T4=theme 4; T5=theme 5; SUI= stress urinary incontinence; UUI= urgency urinary incontinence.

**Table 3:** Participants’ narratives from the theme: Experiencing uncontrolled urinary leakage.

Theme	Thematic Code Groups	Narratives
**Experiencing uncontrolled urinary leakage **	**Unpleasant sensation **	*“The repercussion… well the feeling of always being unclean. (…) Because at times I feel unclean even though I am not even soiled.”* (IU1, 45 years old)
	**Blame **	*“You can’t avoid it, at times I feel guilty. I don’t know, feeling like I have done something to trigger this…”* (IU10, 47 years old)
	**Self-esteem **	*“For people who suffer this, it leads to impressive amounts of stress. You feel like a complete idiot. You are defective… In other words, you feel like you are worthless, because of the fact that you may have a leak… “* (IU2, 45 years old)
	**Fear of body odor**	*“I cannot help it … I’m smelling myself continuously, I’m afraid the smell will be obvious. Sometimes I seem to be crazy, smelling my clothes (laughs)*”(IU15, 49)

**Table 4:** Participants’ narratives from the theme: Information based on Beliefs and Myths Regarding UI.

Theme	Thematic Code Groups	Narratives
**Information based on Beliefs and Myths Regarding UI **	**Lack of information: **	*“You feel disoriented, you don’t know if it is normal or not, whether you should worry or not”* (IU2, 45 years old), *“For chest pain it seems quite clear to me, bleeding also, but for this… where should I go (for help)?” (IU6, 44 years old)*
	**Beliefs & myths: **	*“If you are a woman you know that sooner or later you are going to suffer from it, incontinence”* (IU9,58 years old), *“What happens when you have had children is that the pelvic floor ends up weaker. And although you exercise, it’s normal that after having children you have urine leaks. Whatever you do you can’t avoid it, it’s part of being a mother. This is the message that I have always been given.”* (IU3, 45 years old), *“It’s as if it’s something you inherit, seeing as my grandmother had it, I have to go through it too”* (IU4, 23 years old).

**Table 5:** Participants’ narratives from the theme: Adapting to change and developing strategies.

Theme	Thematic Code Groups	Narratives
**Adapting to change and developing strategies **	**Modifying their habits and routines **	*“It’s not like before, now you have to prepare everything (in advance), the right clothes, extra pads… It changes the way you dress, with whom you go out, when you choose to go out and even where you want to go!”* (IU5, 45 years old), *“I have had a hard time, because as I didn’t want to stop doing it … I was worried that I wasn’t going to be able to hold it in and that although I wore a pad that it was going to leak out more than the pad could absorb, or being somewhere and not being able to get to the bathroom in time… this made me very nervous.”* (IU2, 45 years old)
	**Strategies **	**Strategies**, location of the bathrooms: *“Knowing where the toilet is… If I have to go urgently I don’t have time to ask, as otherwise I may leak, that way I know where it is.”* (IU3, 45 years old), *“I didn’t take long distance busses that had no bathroom. I had to go to university, and where I studied was two hours away from my home, it made me feel very uncomfortable.”* (IU6, 44 years old) ***Strategies, use of sanitary napkins (pads)***: “*I kept wearing the pad, because I was afraid of leaking. I felt more relaxed as I knew that if I leaked it would be ok as I had the pad.”* (IU2, 45 years old) ***Strategies, use of perfume:*** "It´s already a habit, before leaving I spray perfume on my entire body, and also on my clothes. I’d rather people think that I use an exaggerated amount of perfume to smelling any odor of urine.” (UI18, 56 years old)

**Table 6:** Participants’ narratives from the theme: The Role of Education.

Theme	Thematic Code Groups	Narratives
**The Role of Education **	**Family education: **	*“Us mothers should know this to be able to explain it to our daughters. Then our daughters should know how to take care of that part of their body. Teaching them healthy habits regarding hygiene and cleanliness and regarding peeing and pooing. Teaching them that you shouldn’t hold in your pee more than two or three hours. In short… all those things that are quite trifle, but really are habits that help you to keep that part of your body in a good condition.”* (IU2, 45 years old).
	**Urogenital area and sexual education: **	*“It is clear to me that my mother taught me to feel comfortable with my body, to get to know myself, all my parts, this was essential.”* (IU3, 45 years old), *“So of course, if you don’t have information regarding what virginity is, or what the loss of virginity is, or what can happen to you after, then, you can only feel panicky.”* (IU7, 53 years old)
	**Social environment**	*“Society is in debt with women of this generation, we have continued to have children despite our education, based on fear and blame, a lack of support and, many times, incomprehension towards our problems with this private part.”* (IU14, 56 years old).
	**Rediscovering their private parts **	*“… As a girl, I was at a boarding school with nuns (…) we didn’t even know there was an opening different to that for peeing (…) there was no information about the ovaries, the uterus … at 17 years old. And of course, from then on, a great burden… sinful, dangerous…”* (IU9, 58 years old)
	**Emotional burden**	*“You don’t know why, you feel sort of ashamed, you feel embarrassed to talk about it, as if you are somehow a failure, with guilt, you know?”* (IU10, 47 years old),
